# Transgressivity in Key Functional Traits Rather Than Phenotypic Plasticity Promotes Stress Tolerance in A Hybrid Cordgrass

**DOI:** 10.3390/plants8120594

**Published:** 2019-12-12

**Authors:** Blanca Gallego-Tévar, Brenda J. Grewell, Rebecca E. Drenovsky, Jesús M. Castillo

**Affiliations:** 1 Departamento de Biología Vegetal y Ecología, Universidad de Sevilla, Apartado 1095, 41080 Sevilla, Spain; bgallego@us.es; 2 USDA-ARS Invasive Species and Pollinator Health Research Unit, Department. of Plant Sciences, University of California, Mail Stop 4, 1 Shields Avenue, Davis, CA 95616, USA; bjgrewell@ucdavis.edu; 3 Department of Biology, John Carroll University, University Heights, OH 44118, USA; rdrenovsky@jcu.edu

**Keywords:** abiotic stress, halophytes, heterosis, hybridization, salinity, *Spartina*, transgressive segregation

## Abstract

Hybridization might promote offspring fitness via a greater tolerance to environmental stressors due to heterosis and higher levels of phenotypic plasticity. Thus, analyzing the phenotypic expression of hybrids provides an opportunity to elucidate further plant responses to environmental stress. In the case of coastal salt marshes, sea level rise subjects hybrids, and their parents, to longer tidal submergence and higher salinity. We analyzed the phenotypic expression patterns in the hybrid *Spartina densiflora x foliosa* relative to its parental species, native *S. foliosa,* and invasive *S. densiflora*, from the San Francisco Estuary when exposed to contrasting salinities and inundations in a mesocosm experiment. 37% of the recorded traits displayed no variability among parents and hybrids, 3% showed an additive inheritance, 37% showed mid-parent heterosis, 18% showed *best-parent heterosis*, and 5% presented worst-parent heterosis. Transgressivity, rather than phenotypic plasticity, in key functional traits of the hybrid, such as tiller height, conveyed greater stress tolerance to the hybrid when compared to the tolerance of its parents. As parental trait variability increased, phenotypic transgressivity of the hybrid increased and it was more important in response to inundation than salinity. Increases in salinity and inundation associated with sea level rise will amplify the superiority of the hybrid over its parental species. These results provide evidence of transgressive traits as an underlying source of adaptive variation that can facilitate plant invasions. The adaptive evolutionary process of hybridization is thought to support an increased invasiveness of plant species and their rapid evolution.

## 1. Introduction

Climate change, native biodiversity loss, and their concomitant impacts on ecosystems are global environmental changes of paramount international concern for biological conservation, human health, and quality of life on earth [1]. The on-going effects include global warming and sea level rise. In this context, increasing environmental stressors affect plant biodiversity. To avoid extinction, species with dispersal capacity may migrate to more benign habitats, those with the capacity for phenotypic plasticity may adjust in place, whereas adaptive evolution might support persistence in new environmental conditions [2,3]

Hybridization is a powerful evolutionary mechanism that can lead to best-parent heterosis or ‘hybrid vigor’, producing new hybrid phenotypes with higher performance than parental species [4], which can be particularly significant under changing environmental conditions. Hybridization is also a mechanism for rapid evolution of invasiveness in exotic plant species [5,6]. Heterosis is the result of a non-additive phenotypic expression for one or more transgressive hybrid traits [7], which might be genetically and epigenetically controlled [8]. Best-parent heterosis may result in hybrids with extreme phenotypes that have higher fitness under stressful conditions than their parental species [5,9,10]. In this context, natural and artificial hybrid zones may play an important role in maintaining the conservation of genetic variation within and among species under rapidly changing conditions [11,12]. Frequently, the functional trait responses of hybrids that are stress-tolerant can be quite different from trait expression and stress tolerance capacity of parental species [13]. However, sometimes only a few key functional traits of these hybrids may diverge from those of their parental species [14,15].

Phenotypic plasticity is another process that can increase the stress tolerance capacity of hybrids [16,17], which is also adaptive and might be inherited [18,19]. Research has proposed that plant hybrids can be more phenotypically plastic in key functional traits than their parental species as a consequence of modifications in gene expression at the genetic and possibly epigenetic level [20,21]. However, little is known regarding the importance of heterosis and phenotypic plasticity to hybrids that evolve into changing environments. It is important to understand how heterosis, phenotypic plasticity, and their interactions influence stress tolerance and fitness of hybrids when compared to the more limited performance of their parental species. Moreover, our knowledge of how heterosis and phenotypic plasticity respond to the combined effects of multiple stress factors, as is common under natural field conditions, is very limited. Thus, the study of hybrids is an opportunity for documenting the appearance of new phenotypes with adaptive values in response to climate change [22].

The genus *Spartina* (cordgrasses), which is mainly includes polyploid perennial halophytes, provides an ideal model to study the relationships between increasing stress level, transgressivity, phenotypic plasticity, and plant fitness. Hybrids are common between native and exotic cordgrass species [23]. Some of them express best-parent heterosis in some traits [24,25,26], and the allopolyploid *S. anglica* C. E. Hubbard shows high phenotypic plasticity [27]. Additionally, the *Spartina* hybrids in tidal wetland ecosystems are subjected to sea level rise (SLR) as a consequence of current anthropogenic-induced global warming [28]. As a consequence of SLR, *Spartina* hybrids and their parental species are experiencing increased inundation periods, as well as higher tidewater salinity [29,30]. Inundation and salinity are key environmental factors that influence the growth and survival of halophytes [31]. Therefore, it is expected that increasing stress levels on halophytes that result from increasing inundation and salinity regimes will lead to decreases in salt marsh productivity [32,33]. Some studies have analyzed the effects of increasing salinity [34,35,36] or waterlogging [37,38] on hybrids. To our knowledge, the combined effect of salinity and inundation on hybrid phenotypes has not been analyzed from the perspective of heterosis and phenotypic plasticity, although enhanced tolerance has been observed to these individual stressors.

Our study system was the native *Spartina foliosa* Trin., the invasive South American *S. densiflora* Brongn. and their F-1 sterile hybrid *S. densiflora x foliosa* from San Francisco Estuary on the central California coast, USA [23,39]. Both parental species show low genetic diversity in the studied salt marshes [40,41], and their habitats are expected to be substantially impacted by climate change and SLR [30]. We analyzed 36 biochemical, physiological, morphological, and fitness-related traits of the hybrid *S. densiflora x foliosa* in relation to its co-occurring parental species in a mesocosm experiment, in which the three taxa were subjected to the combined effects of salinity, inundation, and their interactions. In a previous study, we analyzed these responses from a functional perspective [42]. In this study, we investigated: (1) the phenotypic expression profile (number of additive and heterotic traits) of *S. densiflora x foliosa* in responses to the combined effects of salinity and inundation; (2) the phenotypic plasticity of the hybrid and its parental species; and, (3) the resulting vegetative and reproductive fitness of the hybrid and both parental species. We hypothesized that the hybrid taxon would present higher fitness than both parental species, due to a complex set of traits showing best-parent heterosis and high phenotypic plasticity. We also predicted that the environmental stress factors would more strongly affect the parental species than their hybrid at extreme levels of salinity and inundation.

## 2. Results

### 2.1. Phenotypic Expression

**Figure 1:**
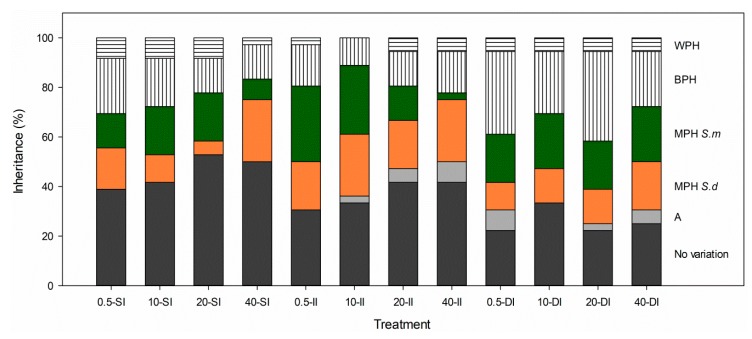
Percentage of phenotypes: same mean values for parents and hybrids (dark gray); additivity (A) (hybrids intermediate between parents; light gray); mid-parent heterosis towards *S. densiflora* (MPH *S.d.*) (orange); mid-parent heterosis towards *S. foliosa* (MPH *S.f.*) (green); best-parent heterosis (BPH) (vertical lines); worst-parent heterosis (WPH) (horizontal lines) for 36 traits of the hybrid *Spartina densiflora x foliosa* at different salinities (0.5, 10, 20 and 40 ppt) and inundation depths (SI, shallow inundation (4.4 cm deep); II, intermediate inundation (35.5 cm deep); DI, deep inundation (55.0 cm deep).

**Figure 2:**
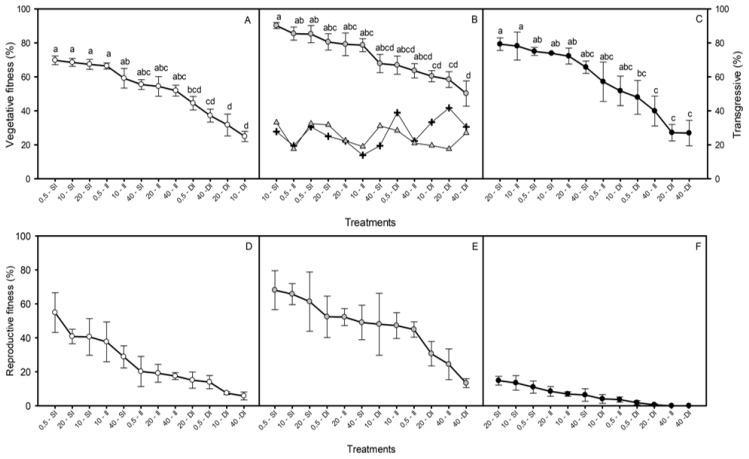
Decreasing percentage of vegetative (**A–C**) and reproductive (**D–F**) fitness (measured as ΔAGB and florets per individual, respectively) of the hybrid *S. densiflora x foliosa* (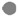) and its parental species *S. densiflora* (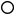), *Spartina foliosa* (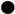) at different salinities (0.5, 10, 20, and 40 ppt) and inundation depths (SI, shallow inundation (4.4 cm deep); II, intermediate inundation (35.5 cm deep); DI, deep inundation (55.0 cm deep)). Values are mean ± SEM (*n* = 4). The percentages of hybrid individuals with transgressive traits (including worst- and *best-parent heterosis)* (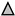) and percentage of traits showing worst- or best-parent heterosis at the hybrid population (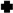) for 36 key functional traits are represented in panel B.

At the population level, the phenotypic expression profile of *S. densiflora x foliosa* to salinity and inundation stress was: 37% of the recorded traits displayed no variability (same mean values for parents and hybrids), 3% showed an additive inheritance (hybrid values intermediate between parental values), 37% showed mid-parent heterosis (MPH), 18% showed best-parent heterosis (BPH), and 5% presented worst-parent heterosis (WPH) (Figure 1; Table S1). The percentage of worst- or best-parent heterosis was the lowest at intermediate salinity and inundation (11% at 10 ppt and II), increasing to both environmental extremes to be c. 30% at freshwater and SI, and at hypersalinity and DI (Figure 1). The percentage of traits and the number of hybrids with worst- or best-parent heterosis did not significantly vary between salinities (two-way ANOVA, salinity: *p* > 0.05; Figure 1, Figure 2B). In contrast, the proportion of traits with worst- or best-parent heterosis was greater under DI (35%) than at II and SI (18 and 24%, respectively) (two-way ANOVA, inundation: *F*_2,11_ = 8.535, *p* < 0.02; Figure 1), whereas the number of hybrids with extreme heterosis traits was higher at SI (34%) than at II and DI (c. 23%) (two-way ANOVA, inundation: *F*_2,11_ = 11.239, *p* < 0.01; Figure 2B).

**Figure 3:**
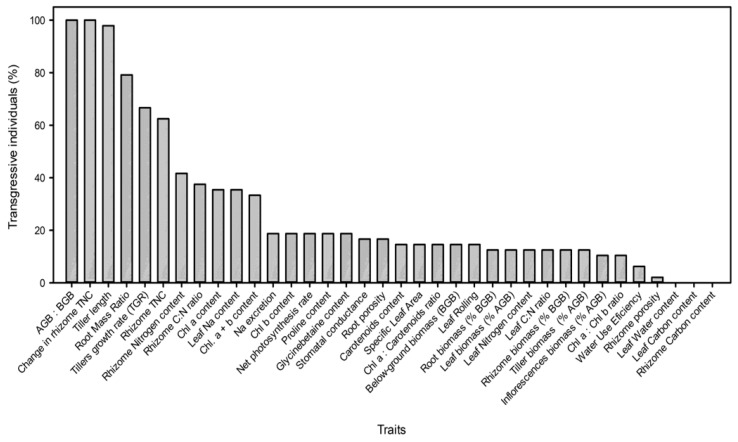
Percentage of individuals of the hybrid *Spartina densiflora x foliosa* showing transgressive traits (including worst- or best-parent heterosis) for 36 morphological, biochemical, and physiological traits measured after exposure to different salinities (0.5, 10, 20, and 40 ppt) and inundation depths (4.4, 35.5, and 55.0 cm deep).

The opposite tendency was found for the hybrid traits showing similar values than both parents that were lower under DI (26%) than at SI (46%) (two-way ANOVA, inundation: *F*_2,11_ = 14.067, *p* < 0.01). The traits for which the majority of hybrids showed BPH were AGB:BGB ratio and change in rhizomes TNC concentration (100%), tillers length (98%), TGR (67%), and rhizomes TNC concentration (63%). On the contrary, no hybrids showed BPH for foliar and rhizome C concentrations and LWC (Figure 3; Table S2).

### 2.2. Plant Trait Variability

**Figure 4:**
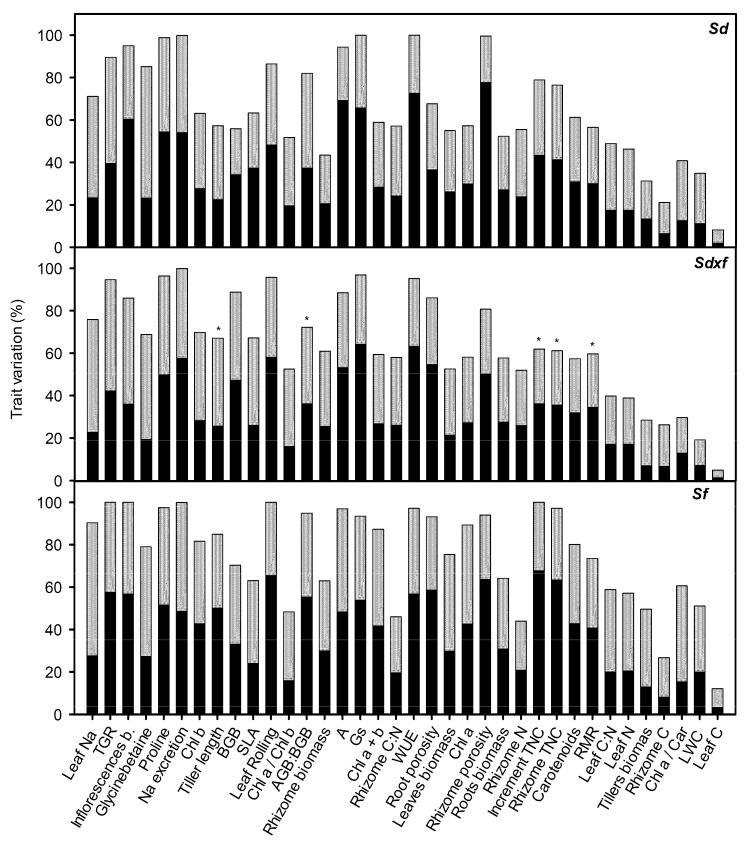
Intra-population trait variability (black), phenotypic plasticity (gray) and inter-treatment trait variability (bar length) for 36 traits measured for *Spartina densiflora* (*Sd*), *S. densiflora x foliosa* (*Sdxf*) and *S. foliosa* (*Sf*) at different salinities (0.5, 10, 20, and 40 ppt) and inundation depths (4.4, 35.5, and 55.0 cm deep). The traits with a transgressive behavior at the population level are marked with an asterisk.

The 36 recorded traits of the hybrid *S. densiflora x foliosa* showed 64 ± 4% of inter-treatment variability, 32 ± 3% of intra-population variability, and 32 ± 2% of phenotypic plasticity in response to the combined effects of salinity and inundation. No significant differences were found between the results for the hybrid and those obtained for its parents for inter-treatment trait variability (*Sf:* 76 ± 4%; *Sd:* 65 ± 4%), for the intra-population trait variability (*Sf:* 38 ± 3%; *Sd:* 34 ± 3%), or phenotypic plasticity (*Sf:* 38 ± 2% > *Sd:* 32 ± 2%; one-Way ANOVA, *F*_2,105_ = 3.478, *p* < 0.05; Figure 4).

The inter-treatment trait variability of the hybrid positively correlated with its phenotypic plasticity and intra-population trait variability (Pearson coefficient, *p* < 0.0001, *n* = 36). The intra-population trait variability of the hybrid was positively correlated to its phenotypic plasticity (*r* = +0.461, *p* < 0.01, *n* = 36). However, intra-population trait variability and phenotypic plasticity were independent of each other for both of the parents. In contrast, the inter-treatment trait variability positively correlated with intra-population trait variability and phenotypic plasticity within the three taxa (Pearson coefficient, *p* < 0.0001, *n* = 36). The traits that were transgressive for a higher number of hybrid plants were those that had greater phenotypic plasticity for *S. densiflora* and higher inter-treatment and intra-population trait variability for *S. foliosa* (Pearson coefficient, *p* < 0.05, *n* = 36). Moreover, the number of hybrid plants with *S. densiflora* dominated traits was negatively related to the phenotypic plasticity of *S. densiflora* (Table 1).

### 2.3. Fitness

The vegetative fitness (change in AGB during the experiment) of the hybrid decreased from 79% to 60% with increasing salinity. Similarly, inundation depth induced a reduction in the vegetative fitness of the hybrid, falling from 80% to 59%. Nonetheless, the hybrid presented greater vegetative fitness than both parental taxa in all of the treatments, except at 10 ppt salinity, where it did not differ from *S. foliosa*. Vegetative fitness fell from 60% in 0.5–20 ppt salinity to 44% in hypersalinity for *S. foliosa* and from 60% in freshwater to 48% in hypersalinity for *S. densiflora*. However, vegetative fitness decreased more with inundation than with salinity in the parental taxa: from 73% to 38% for *S. foliosa* and from 65% to 34% for *S. densiflora* (three-way ANOVA, taxa x salinity: *F*_6,140_ = 2.399, *p* < 0.05; taxa x inundation: *F*_2,140_ = 9.667, *p* < 0.001; Figure 2A–C).

The reproductive fitness (number of florets per plant) of the hybrid was also lower at hypersalinity (29%) than at the rest of the salinities (55%) and it decreased with increasing inundation stress (from 61% to 36%). The reproductive fitness of the hybrid was higher than for both of the parents at every treatment combination. Thus, the decrease of reproductive fitness of the parental species was −13% and −3% with salinity, and −20% and −9% with inundation for *S. densiflora* and *S. foliosa*, respectively (three-way ANOVA, salinity: *F*_2,140_ = 7.110, *p* < 0.001; inundation: *F*_2,140_ = 89.056, *p* < 0.001; taxa: *F*_3,140_ = 25.884, *p* < 0.001; Figure 2D-F). None of the hybrid florets were filled (none contained mature seeds). Figure S1 shows visual differences in fitness at harvests in pictures.

## 3. Discussion

In accordance with our hypothesis, the hybrid *Spartina densiflora x foliosa* showed higher fitness than both parents in relation to hybrid transgressive traits. However, we did not find a complex set of traits showing BPH, counter to our predictions. Instead, we found a small number of key functional transgressive phenotypes that were more strongly expressed at extreme stress levels of inundation than we observed in response to salinity. Another unexpected outcome was that the higher fitness of the hybrid did not result from having greater phenotypic plasticity than its parental species.

Additivity was the most common phenotype of the hybrid. The recorded parental additivity is expected for the F1 hybrids [7,43], but deviations from the mid-parent values can occur if there are non-additive gene expression processes [8,44]. In our study, MPH occurred in 17% of the hybrid traits that were similar to each of two parents. This outcome was likely related to the fact that *S. densiflora x foliosa* receives similar genetic loads from both species (gametes *n* = 35 chromosomes from *S. densiflora* and *n* = 30 chromosomes from *S. foliosa*) [39]. In hybrid maize, the maternal effects on gene expression of hybrid maize were only manifested in reciprocal crosses when the maternal genomic contribution compared to the paternal increased from 1:1 to 2:1 [45]. Similarly, greater maternal effects were observed when the maternal genomic contribution was higher for reciprocal F-1 hybrids between native *Spartina maritima* (Curtis) Fernald and invasive *S. densiflora* in the southwest Iberian Peninsula [26]. F-1 hybrids, as those studied in this work, frequently display maternal effects on gene expression, which promotes different hybrid phenotypes with contrasted stress tolerance [34,46].

Another type of non-additive genetic expression in hybrids is BPH, in which epigenetic processes seem to have a relevant role [47,48,49]. BPH affected 18% of traits of *S. densiflora x foliosa*. Moreover, the number of hybrids with worst- or best-parent heterosis for a given trait increased with the phenotypic plasticity of *S. densiflora* and the intra-population trait variability of *S. foliosa* for that trait. This result highlighted that parental trait variability (expressed within populations at the same environmental conditions or due to environmental changes) might determine the heterosis [26]. This relationship between the heterosis of hybrids and the phenotypic diversity of their parents may be a result of large epigenetic changes recorded after the hybridization between *Spartina* taxa [48], since phenotypic plasticity and heterosis are both regulated at the epigenetic level [21].

We expected that the phenotypic plasticity of the hybrid would be higher than those of its parental species, since genetic and epigenetic changes that occur during hybridization may promote phenotypic plasticity [17]. However, the phenotypic plasticity of *S. densiflora x foliosa* (33%) was similar to both of its parents, with *S. foliosa* presenting a greater value (38%) than *S. densiflora* (32%). The three *Spartina* taxa are polyploids, which may lead to increased phenotypic plasticity [16,50]. *S. foliosa* was the taxon with the lowest ploidy level (*S. foliosa* 2n = 60, *S. densiflora* 2n = 70, hybrid 2n = 65) and it presented the greatest phenotypic plasticity. Part of the high plasticity recorded for *S. foliosa* was passive [51], as reflected in the greater decrease in its vegetative fitness with increasing abiotic stress. In fact, the traits that showed higher plasticity for *S. foliosa*, exceeding those values of *S. densiflora* and the hybrid, were due to the high accumulation of Na in leaves, low net photosynthetic rates, low percentage of leaves in AGB, and low chlorophyll concentration under high levels of combined abiotic stresses.

The greater vegetative and reproductive fitness of the hybrid in relation to both parents recorded in all treatments (except for similar vegetative fitness than *S. foliosa* at 10 ppt salinity) coincided with the abundance of positive heterosis. Most of the *Spartina* hybrid individuals (> 50%) presented BPH due to producing more and taller tillers than its parents, which resulted in high AGB: BGB and low RMR. This resulted in an extreme phenotype relative to the parental species. In addition, most transgressive hybrids also showed high rhizome TNC concentration that increased during the experiment. Tall tillers would be advantageous to a hybrid phenotype in anoxic, waterlogged conditions, since it maintains a greater proportion of biomass above the water surface, facilitating greater access to light and CO_2_ that drive growth [52,53]. This morphology would also support greater oxygen diffusion to roots under anoxic conditions [54]. Under these conditions, greater photosynthetic biomass over the water surface supports higher growth rates and higher subterranean energy reserves (rhizome TNC) to supply demand under stressful conditions [55]. Thus, tall tillers would be especially important in the maintenance of fitness under permanent flooding conditions that are expected in *Spartina* habitat with SLR. The positive heterosis that resulted in tall tillers might be due to combinations of parental alleles with dominance effects or a response to disturbance related epistatic interactions [34]. We did not observe any BPH for key biochemical and physiological traits, in contrast with the large, recorded changes in morphological and biomass-allocation traits in the hybrid. In this sense, heterosis is maximized when the parents are phenotypically close and when the distributions of parental traits are contrasted, constrained [56], and show nonlinear relationships between them [57].

The hybrid reduced its vegetative and reproductive fitness with increasing stress less than its parents, whose fitness was more affected by inundation depth than by salinity, s a consequence of the recorded positive heterosis in key morphological traits. The BPH that was recorded for some hybrids at extremes of experimentally-imposed stress drivers was due to parental species experiencing high negative effects in those stress conditions. For example, *S. densiflora* often has optimal physiological responses under intermediate brackish salinity conditions, rather than at freshwater or hypersalinity [58,59]. In *Spartina,* different genotypes of *S. densiflora x foliosa* have previously outperformed both parental taxa by producing taller tillers at high salinities [36]. The same has been observed for the responses of reciprocal hybrids between *S. maritima* and *S. densiflora* in response to salinity [25,26]. Our results characterizing just one trait with positive heterosis, tall tillers, as a key plant characteristic that clearly supports tolerance to inundation, contrasts with previous studies that have associated high fitness of plant hybrids to a complex suite of superior physiological and morphological traits [60]. Nevertheless, the documentation of entirely opposite functional trait responses when hybrid plants are exposed to both salinity and inundation would limit the tolerance to the interaction between these stress factors and result in intermediate and non-optimal responses for either of the interacting stresses [61]. Thus, these contradictory responses, which were more abundant for the hybrid than for its parents, can partially counteract the positive heterosis.

It is remarkable that the highest numbers of traits showing BPH were observed for only a few individual hybrid plants that were subjected to deep inundation. This suggests that relatively few individuals had a high number of unique traits expressing BPH, likely because each hybrid was the product of independent hybridization events. *Spartina densiflora x foliosa* has been reported as sterile [36,39], which was also observed in our study, given the absence of mature seeds inside florets. Fertility can be developed in sterile F1 hybrids if the process of interspecific hybridization is followed by a chromosomal duplication originating a new allopolyploid species [62,63]. This process has already been observed in the genus *Spartina* for the allopolyploid *Spartina anglica* in Europe [27,64]. According to our results, if chromosomal doubling occurs in the genome of *S. densiflora x foliosa,* the resulting allopolyploid would likely have the capacity to aggressively spread via sexual propagules at a rate potentially much greater than seed dispersal and colonization that was observed for its invasive parent *S. densiflora* [25,65,66] at a wide range of salinities and inundation regimes.

## 4. Materials and Methods

### 4.1. Experimental Design

All of the plants were collected in July–November 2016 from middle tidal marshes in the San Francisco Estuary (California, USA). The plants of *Spartina foliosa* (2n = 6x = 60) were collected in the Carquinez Straits (38°3′57″ N, 122°11′36″ W), and *S. densiflora* (2n = 7x = 70) and the hybrid *S. densiflora x foliosa* (2n = x6.5 = 65) were collected from tidal wetlands along Corte Madera Creek (37°56′27″ N, 122°31′2″ W). The ploidy level of the hybrids was determined previous to the beginning of the experiment by somatic chromosome counts [67,68], with modifications adapted from Singh [69] to confirm precise study taxa identification. 

The rhizomes were separated from aerial tillers and roots, and cleaned to obtain similar-sized experimental individuals, according to the growth form of each taxon, at the USDA-ARS Aquatic Weed Research Facility, Agricultural Experiment Station, University of California, Davis, USA. On 3 March 2017, the rhizomes were transplanted to pots (15.0 cm diameter x 17.5 cm height) with bottom drainage holes, while using sterile sand as substrate, and grown until the beginning of the experiment. The base of the pots was kept flooded to sub-irrigate with freshwater (5 cm depth), and nutrients were added as 10 mL of 40% Hoagland’s nutrient solution pipetted onto the sediment surface of each pot once per week. The plants of each taxon were randomly assigned to experimental treatments. The higher saline treatments were obtained by gradually increasing the salinity concentration of the solution by 10 ppt per week until the target experimental salinity level was achieved to avoid osmotic shock in the plants. After salinity conditioning, on 8 May 2017, the plants were arranged in a split-plot, full factorial, randomized complete block design, in which the salinity factor was randomly assigned to mesocosms as the main plot. The treatments were nested within sixteen 500 L (1.3 m x 0.8 m x 0.6 m) plastic mesocosms (Rubbermaid, Atlanta, GA, USA). The factor inundation depth was assigned to the subplots with each of three taxa (*n* = 4 plants per treatment) nested within each subplot (Figure S2). The salinity treatments ranged from freshwater to hypersalinity (0.5, 10, 20 and 40 ppt) and they were created while using sea salt Instant Ocean^®^ (Aquarium Systems Inc., Mentor, OH, USA) plus 20% Hoagland’s nutrient solution and Eco Pond Clear biological product (Grow More Inc., Gardena, CA, USA) for reducing algae proliferation. Three permanent inundation treatments (shallow inundation (SI: 4.4 cm deep from the base of the pots), intermediate inundation (II: 35.5 cm deep), and deep inundation (DI: 55.0 cm deep)) were established by placing the plants on top of concrete block platforms at different heights inside each tank. The glasshouse conditions were maintained with controlled air temperature (21–25 °C) and a 12 h daily photoperiod set between 500 and 1500 μmol m^−2^ s^−1^ at the canopy level with high intensity discharge lights (GE Lucalox LU1000/ ECO HPS 1000 W, PARsource, Petaluma, CA, USA). The experiment was carried out for 44 days until harvest.

### 4.2. Plant Traits

A suite of 36 biochemical, physiological, morphological, and fitness-related traits were recorded for the three studied taxa to obtain a representative phenotypic expression profile of the hybrid. Measurements of net photosynthesis rate (*A*) and stomatal conductance (*g_s_*) were carried out while using a LI-COR 6400 portable infrared CO_2_ analyzer (LI-COR Biosciences, Lincoln, NE, USA) in differential mode and in an open circuit. The CO_2_ concentration inside the chamber was fixed at 400 μmol mol^−1^, photon flux density was 1000 μmol m^−2^ s^−1^ (actinic light source 90% red and 10% blue), and flow rate 400 μmol s^−1^. Before taking each measurement, one leaf per plant was wiped with a tissue wet with distilled water to remove the salt accumulated on the surface and left to dry. Each individual measurement was the mean of three subreplicates separated by a 10-s interval. All of the measurements were conducted on sunny days (13–17 June 2017) within around solar noon. Intrinsic water use efficiency (WUE) was calculated as the ratio of *A* to *g_s_* [70].

The porosity of roots and rhizomes was determined following Kercher and Zedler [71], while using fragments of live roots and rhizomes of 5 cm length. Porosity (%) was calculated as the difference between initial and final weight in relation to the initial weight.

All of the leaf measurements were conducted on flag leaves (first unfolded adult leaf from the apical leaf) to avoid effects due to the leaf ontogeny. Leaf adaxial rolling was calculated as the percentage reduction in leaf width after rolling [72]. Specific leaf area (SLA, m^−2^ g^−1^) was determined for three leaves per plant as the ratio between the leaf area and its dry weight (DW) [73]. Leaf area calculated while using WinFOLIA (Regent Instruments Inc., Saint-Foy, Quebec, Canada) and DW was assessed after oven-drying the leaves at 70 °C for 48 h. The Leaf Water Content (LWC) was obtained for one leaf per plant and LWC was calculated on the total leaf mass of the entire plant as LWC (%) = (FW − DW) × 100 / FW, where FW is the fresh weight [74].

Fresh leaf tissue (0.5 – 2.0 g per plant) was collected and immediately frozen for later analyses. The determination of chlorophyll *a* (Chl *a*), chlorophyll *b* (Chl *b*), and carotenoids (Car) concentration was carried out following Lichtenthaler [75] and while using a spectrophotometer (Beckman DU-64, Beckman Coulter, Inc., Brea, CA, USA). Additionally, dry leaf tissue was ground to pass through a No. 40 mesh screen for nitrogen (N), carbon (C), and sodium (Na). The total leaf N and C content were determined using a Perkin Elmer 2400 CHNS/O analyzer (Perkin Elmer, Waltham, MA, USA). The ratio C:N was calculated. Leaf Na was measured using a sodium electrode on dry-ashed samples that were dissolved in 1 M HCl [57]. Free proline content in leaves was determined following Bates et al. [76]. The glycinebetaine content in leaves was estimated as quaternary ammonium compounds following Grieve and Grattan [77]. The leaf sodium (Na) exudation rate was measured following Swank et al. [78]. The total content of C, N, and the C:N index of rhizomes was obtained, as reported above for leaves. The rhizomes were analyzed for total non-structural carbohydrates (TNC) while using a modified enzymatic digestion procedure, as detailed by Swank et al. [79], followed by spectrophotometric assay for reducing sugars [79]. 

At the end of the experiment, the below-ground biomass (BGB) of each tussock was separated into roots and rhizomes, and the above-ground biomass (AGB) into leaves, tillers, and inflorescences. Dry weights (g) were obtained after oven-drying at 70 °C for 48 h. The proportion (%) of each organ in relation to BGB or AGB and the AGB:BGB ratio was calculated [80]. Root mass ratio (RMR) was also calculated as the proportion of roots (DW) in relation to the total biomass [81]. The number of tillers (live, dead, and flowered) was counted for each clump at the beginning and at the end of the experiment. Tiller relative growth rate (TGR, tillers tillers^−1^ yr^−1^) was calculated as the difference between the number of final and initial tillers, divided by the number of experiment days [25]. The lengths of tillers were measured for five adult tillers of each tussock at the end of the experiment. The number of florets per plant was determined from the average number of florets per inflorescence for each taxa at each treatment (*n* = 1 inflorescence per plant) and the number of inflorescences per individual (*n* = 4 plants per treatment). 

### 4.3. Phenotypic Expression

The phenotypic expression for the 36 recorded plant traits in responses to salinity and inundation was recorded for the hybrid *S. densiflora x foliosa*. Four different phenotypic expressions were determined at the population level for each plant trait and treatment combination (*n* = 12): 1) The additivity (A) when the trait value for the hybrid was equal to the average parental value; 2) Worst-parent heterosis (WPH) when the hybrid value was lower than the worst parental value; 3) Mid-parent heterosis (MPH) when the hybrid value was lower or higher than the mean parental phenotype, but higher than the worst (negative heterosis) and lower than the best (positive heterosis) parental value; and, 4) Best-parent heterosis (BPH) when the hybrid value was higher than the best parental value [82]. In all cases, the mean heterosis values were quantified, since the hybridizations occurred between parental populations. At the individual level, the number of hybrids with best-parent heterosis was computed for each plant trait and treatment combination.

### 4.4. Plant Trait Variability

Inter-treatment plant trait variability index was calculated for each taxon as the relation of the difference between the maximum (X_max_) and the minimum (X_min_) values of a given plant trait divided by its maximum, when all of the values of the different treatment combinations were included [83]. Inter-treatment trait variability index was considered as a general indicator of trait variability among treatments and individuals for each taxon.

(1) $$\text{Inter. Trait Var.}=\frac{\left(X_{\max}-X_{\min}\right)\times 100}{X_{\max}}$$

Mean intra-population trait variability index was calculated for each taxon as the arithmetic average (*n* = 12 inundation x salinity treatments) of the relation between the difference between the maximum (x_max_) and the minimum (x_min_) values divided by the maximum of a given plant trait at each salinity x inundation combination [40]. The intra-population trait variability was used as an intrinsic indicator of trait variation within populations.

(2) $$\text{Intra. Trait Var.}=\frac{\sum_{i=1}^{12}\frac{\left(X_{\max}-X_{\min}\right)\times 100}{X_{\max}}}{12}$$

The phenotypic plasticity index (PPI) was determined after subtracting the intra-population component to the inter-treatment trait variability [26], since intra-population variations in plant traits may be important due to the differences between individuals in polyploid taxa such as *Spartina* [84] and especially in sterile hybrids [50]. Thus, the trait variability in PPI was only related to the responses to the application of the inundation and salinity treatments.

### 4.5. Fitness

Reproductive and vegetative fitness were measured for the three *Spartina* taxa at the different treatment combinations as the mean percentage in relation to the maximum value of fitness-related traits. Vegetative fitness was calculated while using the change (Δ) in AGB [85], while the total number of florets produced per individual was used for reproductive fitness [25]. Fitness was calculated, as follows:

(3) $$\mathrm{Fitness}\;(\%)=\frac{X_{i}\times 100}{X_{\max}}$$

where x_i_ was the value of ΔAGB or the total number of florets per individual for the treatment combination i and x_max_ was the maximum value for that fitness trait at every treatment combination. The florets were pressed with a fingertip to check if they contained any seeds inside in order to evaluate the production of mature seeds of the hybrids [25]. The percentage of change in absolute high and low mean trait values is a suitable method for quantifying phenotypic plasticity *sensu lato*, since it allows for comparing values among different traits and taxa with divergent ranges in variation and/or that are expressed in different units [83].

### 4.6. Statistical Analyses

All of the statistical analyses were performed with IBM SPSS V. 20 for Windows while using a significance level (α) of 0.05. All data were evaluated for normality, and the homogeneity of variances of all the data sets were tested while using Levene’s Test, performing transformations of square root (for BGB and Na excretion rate) and 1 / x (for SLA, leaf C:N ratio, leaf Na, proline, *g_s_*, WUE, and rhizome C: ratio) if homoscedasticity was not reached. The main univariate differences of hybrid traits to salinity and inundation and their relationships to its parental species were assessed while using General Lineal Models (GLMs) and Bonferroni–Dunn’s test as post hoc analysis. We evaluated the significance of the factors salinity (0.5, 10, 20, and 40 ppt) and inundation depth (shallow, intermediate, and deep inundation) for the hybrid, and we added the taxon factor (*S. densiflora x foliosa, S. densiflora* and *S. foliosa*) to determine the different phenotypic expressions. Two-way analysis of variance (ANOVA) with salinity and inundation as grouping factors were carried out to compare the average number of transgressive traits and hybrid individuals with transgressive responses as dependent variables. The means of trait variability indexes, and vegetative and reproductive fitness were compared between treatments and taxa while using three-way ANOVA with taxon, inundation depth, and salinity as the grouping factors. The relationships between intra-population and inter-treatment trait variability and phenotypic plasticity indexes for the different traits of each taxon were calculated while using Pearson correlation coefficient (r).

## 5. Conclusions

The increased stress tolerance through transgressive phenotypes of the hybrid *S. densiflora x foliosa*, which increased together with the level of trait variability of its parents, led to relatively high vegetative and reproductive fitness, even under extreme stress imposed by high salinity and inundation conditions. The development of a key transgressive trait, tall tillers, under high inundation stress was especially important, which led to reduced fitness of both native and exotic parental species. The sterility of this extreme phenotypic hybrid is currently a limitation to its capacity for spread. However, the chromosomal doubling of the polyploid yielding fertile hybrids, coupled with SLR-driven increases in tidal inundation and salinity, might promote the fitness and invasiveness of the hybrid over its native and exotic parental species over time.

## Figures and Tables

**Table 1:** Pearson correlation coefficients and *p*-values between intra-population (Intra) and inter-treatment (Inter) trait variability and phenotypic plasticity (PP) of *S. densiflora x foliosa* (*Sdxf*), *S. densiflora* (*Sd*) and *S. foliosa* (*Sf)*; and the number of hybrid individuals with transgressive phenotype (T), and *S. densiflora* (D-Sd) and *S. foliosa* (D-Sf) dominant phenotype for 36 traits measured at different salinities (0.5, 10, 20 and 40 ppt) and inundation depths (4.4, 35.5, and 55.0 cm deep). Significant differences (*p* < 0.05) are marked in bold.

**Intra-Population Trait Variability**			***S. densiflora x foliosa***						
	*Sd*	*Sdxf*		Intra	PP		T	D-Sd	D-Sf
*Sd*	**0.794**	**0.89**	Inter	**0.911**	**0.785**	Inter (*Sd*)	0.193	−0.0899	0.112
	**<0.0001**	**<0.0001**		**<0.0001**	**<0.0001**		0.26	0.602	0.514
									
*Sf*		**0.817**	Intra		**0.461**	Intra (*Sd*)	0.0293	0.0756	0.16
		**<0.0001**			**<0.0001**		0.865	0.661	0.352
									
Inter-Treatment Trait Variability			*S. densiflora*			PP (*Sd*)	**0.384**	**−0.34**	−0.0336
	*Sd*	*Sdxf*		Intra	PP		**0.0207**	**0.0424**	0.846
*Sd*	**0.874**	**0.9**	Inter	**0.904**	**0.636**				
	**<0.0001**	**<0.0001**		**<0.0001**	**<0.0001**	Inter (*Sf*)	**0.357**	−0.178	0.0249
							**0.0327**	0.299	0.885
*Sf*		**0.83**	Intra		0.245				
		**<0.0001**			0.15	Intra (*Sf*)	**0.422**	−0.269	−0.0366
							**0.0103**	0.112	0.832
Phenotypic Plasticity			*S. foliosa*						
	*Sd*	*Sdxf*		Intra	PP	PP (*Sf*)	0.0532	0.0826	0.127
*Sd*	**0.643**	**0.722**	Inter	**0.914**	**0.653**		0.758	0.632	0.46
	**<0.0001**	**<0.0001**		**<0.0001**	**<0.0001**				
						Inter (*Sdxf*)	0.159	−0.0795	0.122
*Sf*		**0.652**	Intra		0.29		0.354	0.645	0.479
		**<0.0001**			0.086				
						Intra (*Sdxf*)	0.103	−0.0395	0.162
							0.548	0.819	0.346
									
						PP (*Sdxf*)	0.188	−0.112	0.0198
							0.273	0.516	0.909

## Supplementary Materials

The following are available online at https://www.mdpi.com/2223-7747/8/12/594/s1, Table S1: Phenotypic expressions (parental dominance (D); parental additivity (I); transgressive (T)) for the hybrid *Spartina densiflora x foliosa* (*Sdxf*) for 36 traits measured at different salinities (0.5, 10, 20 and 40 ppt) and inundation depths (shallow inundation (SI), 4.4 cm; Intermediate inundation (II), 35.5 cm; Deep inundation (DI), 55.0 cm deep). Parental taxa: *S. densiflora* (*Sd*); *S. foliosa* (*Sf*). Three-way ANOVA, salinity *x* inundation *x* taxa, *p* < 0.05, *n* = 4; Table S2: Number of individuals of *Spartina densiflora x foliosa* (*n* = 4) with transgressive response for 36 traits measured at each treatment combination of salinities (0.5, 10, 20 and 40 ppt) and inundation depths (shallow inundation (SI), 4.4 cm deep; intermediate inundation (II), 35.5 cm deep; deep inundation (DI), 55.0 cm deep). Percentages of individuals are marked in bold. Figure S1: Pictures of individuals of *Spartina foliosa*, *S. densiflora x foliosa* and *S. densiflora* (from left to right for each inundation treatment) after 44 days under shallow (SI, 4.4 cm deep), intermediate (II, 35.5 cm deep) and deep inundation (DI, 55.0 cm deep) and exposed to (A) 0.5 ppt salinity and (B) 40 ppt salinity.
