# Supplementary material for: Transgressivity in Key Functional Traits Rather Than Phenotypic Plasticity Promotes Stress Tolerance in A Hybrid Cordgrass
Source: Plants (Basel). 2019 Dec 12;8(12):594. doi: 10.3390/plants8120594 (PMC6963473; doi:10.3390/plants8120594)
Supplement: Supplementary file 1 [file plants-08-00594-s001.zip › Figure_S1.pdf]

Gallego-Tévar et al. - Plants - *Supplementary Materials* Figure S1

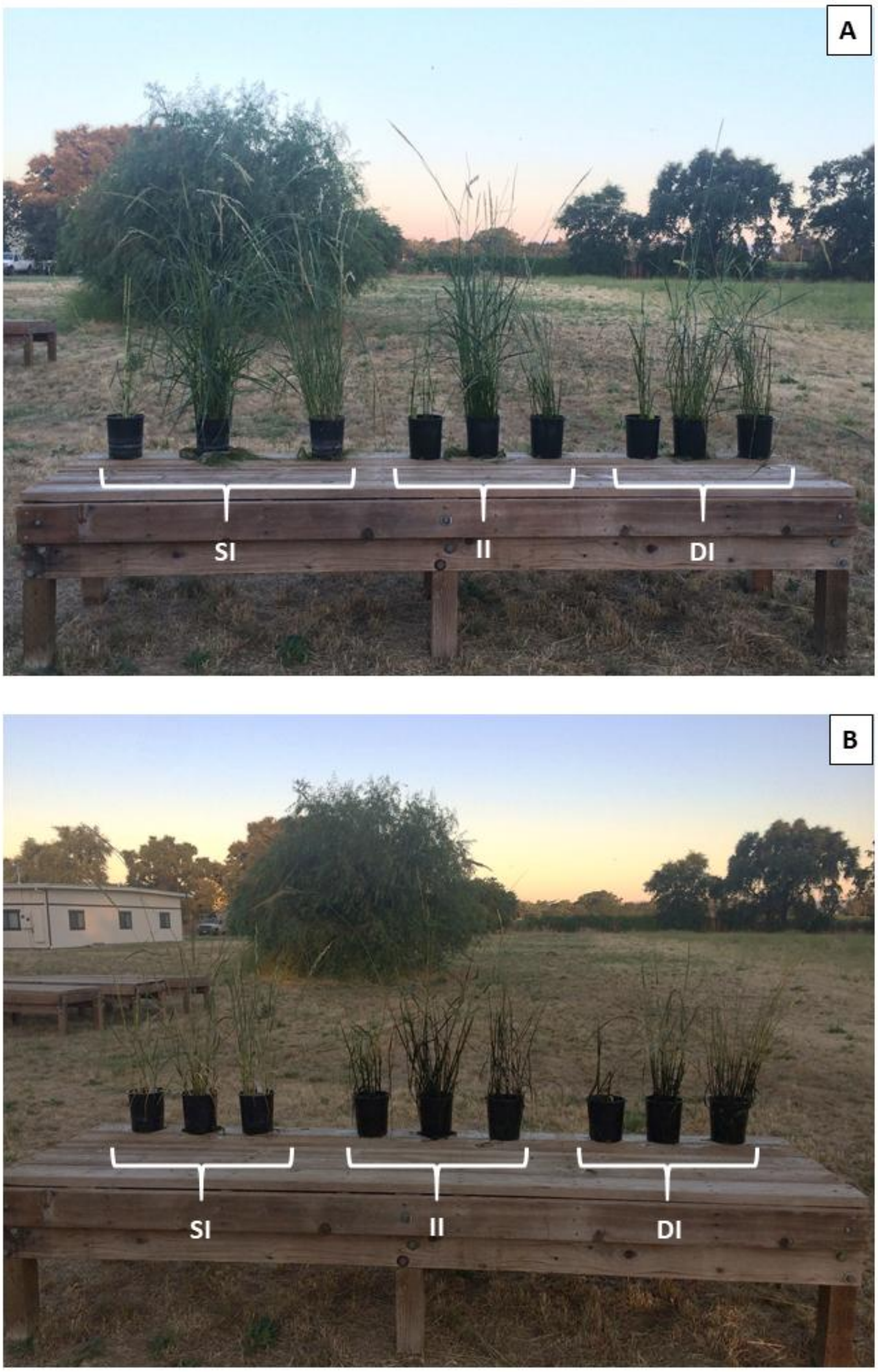

Figure S1. Pictures of individuals of *Spartina foliosa*, *S. densiflora x foliosa* and *S. densiflora* (from left to right for each inundation treatment) after 44 days under shallow (SI, 4.4 cm deep), intermediate (II, 35.5 cm deep) and deep inundation (DI, 55.0 cm deep) and exposed to (A) 0.5 ppt salinity and (B) 40 ppt salinity.
