# Supplementary material for: Transgressivity in Key Functional Traits Rather Than Phenotypic Plasticity Promotes Stress Tolerance in A Hybrid Cordgrass
Source: Plants (Basel). 2019 Dec 12;8(12):594. doi: 10.3390/plants8120594 (PMC6963473; doi:10.3390/plants8120594)
Supplement: Supplementary file 1 [file plants-08-00594-s001.zip › Figure_S2.pdf]

plants MDPI

Gallego-Tévar et al. - Plants - *Supplementary Materials* Figure S2

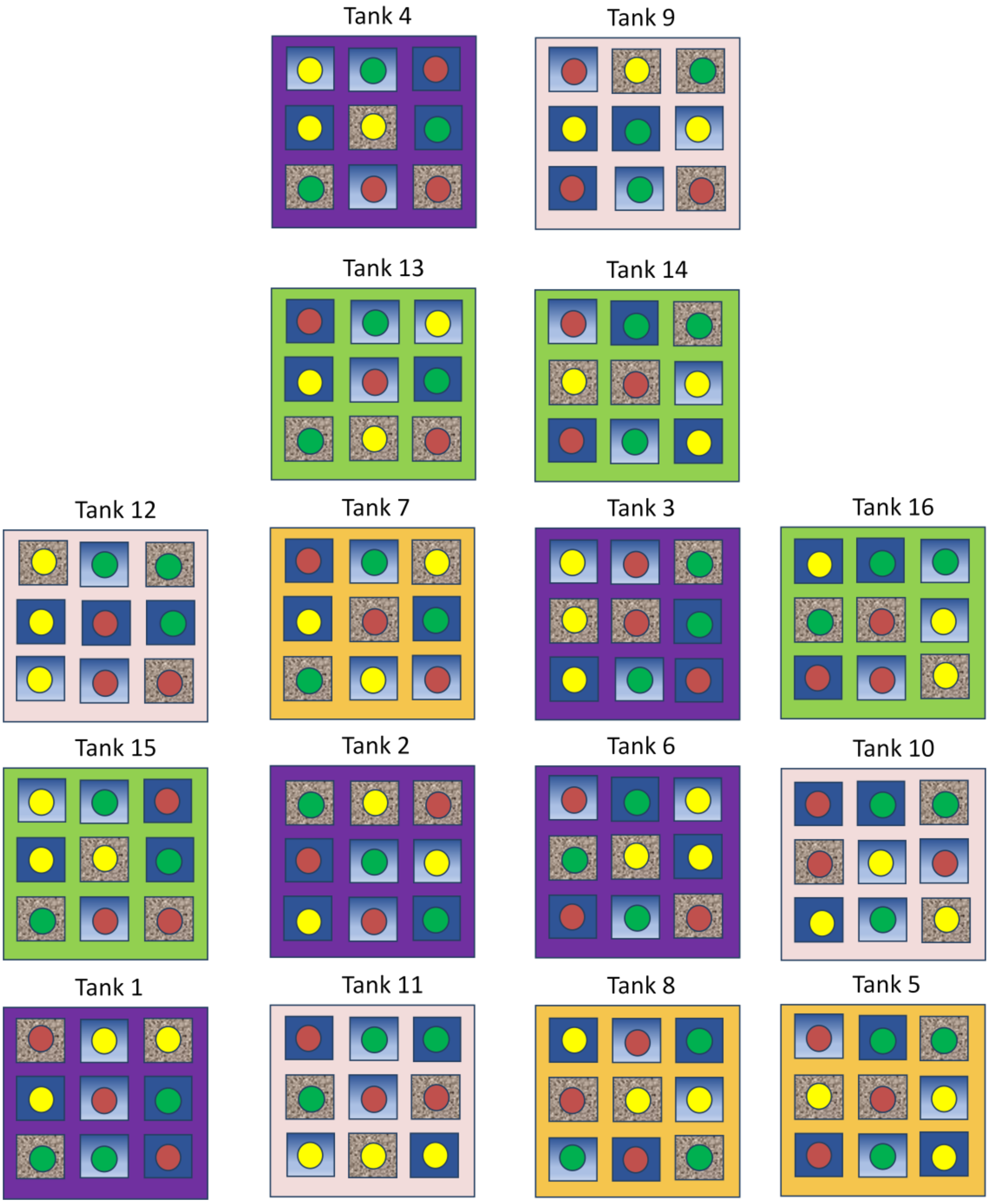

Figure S2. Split-plot, full factorial experiment design in which salinity was assigned as the main plot in a randomized complete block design, inundation was assigned to the subplots, and the three taxa (n = 4 plants per treatment) were nested within each subplot. Salinity treatments (large plots): 0.5 (yellow), 10 (purple), 20 (ocher) and 40 ppt (green). Inundation treatments (small plots): shallow (doted brown), intermediate (dark blue), deep (dark to light blue). *Spartina* taxa (circles): *S. foliosa* (orange), *S. densiflora* (green), *S. foliosa x densiflora* (yellow).
