# Supplementary material for: Transgressivity in Key Functional Traits Rather Than Phenotypic Plasticity Promotes Stress Tolerance in A Hybrid Cordgrass
Source: Plants (Basel). 2019 Dec 12;8(12):594. doi: 10.3390/plants8120594 (PMC6963473; doi:10.3390/plants8120594)
Supplement: Supplementary file 1 [file plants-08-00594-s001.zip › Table_S1.pdf]

Gallego-Tévar et al. - Plants - *Supplementary Material* Table S1.

Table S1. Phenotypic expressions (additivity being equal to both parental species (*Sd-Sf*); additivity being intermediate between parents (A); mid-parent heterosis (MPH); best-parent heterosis (BPH); worst-parent heterosis (WPH)) for the hybrid *Spartina densiflora x foliosa* for 36 traits measured at different salinities (0.5, 10, 20 and 40 ppt) and inundation depths (shallow inundation (SI), 4.4 cm; Intermediate inundation (II), 35.5 cm; Deep inundation (DI), 55.0 cm deep). Parental taxa: *S. densiflora* (*Sd*); *S. foliosa* (*Sf*). Three-way ANOVA, salinity *x* inundation *x* taxa, $P < 0.05$, $n = 4$.

	**0.5 ppt**			**10 ppt**			**20 ppt**			**40 ppt**		
	**SI**	**II**	**DI**	**SI**	**II**	**DI**	**SI**	**II**	**DI**	**SI**	**II**	**DI**
AGB : BGB	BPH	BPH	BPH	BPH	BPH	BPH	BPH	BPH	BPH	BPH	BPH	BPH
Below-ground biomass (BGB)	MPH	MPH	A	BPH	MPH	MPH	MPH	A	A	MPH	A	MPH
Carotenoids content	*Sd-Sf*	MPH	BPH	*Sd-Sf*	MPH	MPH	*Sd-Sf*	MPH	BPH	*Sd-Sf*	*Sd-Sf*	MPH
Chl *a* : Carotenoids ratio	MPH	MPH	BPH	*Sd-Sf*	*Sd-Sf*	MPH	*Sd-Sf*	*Sd-Sf*	BPH	*Sd-Sf*	MPH	MPH
Chl *a* : Chl *b* ratio	*Sd-Sf*	MPH	MPH	*Sd-Sf*	MPH	MPH	*Sd-Sf*	MPH	MPH	*Sd-Sf*	MPH	MPH
Chl *a* content	*Sd-Sf*	MPH	BPH	*Sd-Sf*	A	BPH	*Sd-Sf*	MPH	BPH	MPH	*Sd-Sf*	BPH
Chl *b* content	*Sd-Sf*	MPH	BPH	*Sd-Sf*	MPH	MPH	*Sd-Sf*	*Sd-Sf*	BPH	*Sd-Sf*	*Sd-Sf*	*Sd-Sf*
Chl. *a* + *b* content	*Sd-Sf*	MPH	BPH	*Sd-Sf*	MPH	BPH	*Sd-Sf*	MPH	BPH	MPH	*Sd-Sf*	BPH
Glycinebetaine content	BPH	MPH	MPH	MPH	MPH	MPH	MPH	*Sd-Sf*	MPH	MPH	MPH	*Sd-Sf*
Inflorescences biomass (% AGB)	*Sd-Sf*	*Sd-Sf*	*Sd-Sf*	*WPH*	MPH	*Sd-Sf*	*Sd-Sf*	*Sd-Sf*	MPH	*Sd-Sf*	A	A
Leaf biomass (% AGB)	MPH	MPH	MPH	MPH	*Sd-Sf*	MPH	*Sd-Sf*	*Sd-Sf*	*WPH*	MPH	MPH	*Sd-Sf*
Leaf C:N ratio	*WPH*	MPH	MPH	MPH	MPH	*Sd-Sf*	*Sd-Sf*	MPH	*Sd-Sf*	MPH	MPH	MPH
Leaf Carbon content	MPH	MPH	MPH	MPH	MPH	BPH	MPH	MPH	BPH	MPH	MPH	BPH
Leaf Na content	*WPH*	MPH	A	*Sd-Sf*	MPH	MPH	*WPH*	MPH	MPH	MPH	MPH	MPH
Leaf Nitrogen content	BPH	MPH	MPH	MPH	MPH	MPH	*Sd-Sf*	*Sd-Sf*	BPH	*Sd-Sf*	MPH	MPH
Leaf Rolling	MPH	MPH	MPH	MPH	MPH	*Sd-Sf*	MPH	MPH	MPH	BPH	*Sd-Sf*	MPH
Leaf Water content	*Sd-Sf*	*Sd-Sf*	*Sd-Sf*	*Sd-Sf*	*Sd-Sf*	*Sd-Sf*	*Sd-Sf*	*Sd-Sf*	*Sd-Sf*	*Sd-Sf*	*Sd-Sf*	*Sd-Sf*
Na excretion	*Sd-Sf*	*Sd-Sf*	*Sd-Sf*	*Sd-Sf*	MPH	BPH	*Sd-Sf*	MPH	MPH	MPH	A	MPH
Net photosynthesis rate	MPH	MPH	*Sd-Sf*	MPH	*Sd-Sf*	*Sd-Sf*	MPH	*Sd-Sf*	*Sd-Sf*	*Sd-Sf*	MPH	*Sd-Sf*
Proline content	BPH	*Sd-Sf*	A	*Sd-Sf*	*Sd-Sf*	*Sd-Sf*	*Sd-Sf*	MPH	*Sd-Sf*	*Sd-Sf*	*Sd-Sf*	*Sd-Sf*
Rhizome biomass (% BGB)	*Sd-Sf*	*Sd-Sf*	*WPH*	*Sd-Sf*	MPH	*Sd-Sf*	MPH	*Sd-Sf*	MPH	*Sd-Sf*	*Sd-Sf*	*WPH*
Rhizome C:N ratio	*WPH*	MPH	MPH	*WPH*	MPH	MPH	*WPH*	*WPH*	MPH	MPH	*WPH*	MPH
Rhizome Carbon content	*Sd-Sf*	MPH	MPH	MPH	*Sd-Sf*	*Sd-Sf*	*Sd-Sf*	*Sd-Sf*	MPH	MPH	*Sd-Sf*	MPH
Rhizome Nitrogen content	BPH	BPH	MPH	BPH	MPH	MPH	BPH	BPH	MPH	MPH	BPH	MPH
Rhizome porosity	MPH	*Sd-Sf*	*Sd-Sf*	MPH	*Sd-Sf*	MPH	*Sd-Sf*	A	*Sd-Sf*	*Sd-Sf*	*Sd-Sf*	MPH
Rhizome TNC	*Sd-Sf*	BPH	BPH	BPH	BPH	BPH	BPH	BPH	BPH	BPH	BPH	BPH
Root biomass (% BGB)	*Sd-Sf*	*Sd-Sf*	BPH	*Sd-Sf*	MPH	*Sd-Sf*	MPH	*Sd-Sf*	MPH	*Sd-Sf*	*Sd-Sf*	BPH
Root Mass Ratio	MPH	*WPH*	*WPH*	*WPH*	MPH	*WPH*	*WPH*	*WPH*	*WPH*	*WPH*	*WPH*	*WPH*
Root porosity	MPH	*Sd-Sf*	*Sd-Sf*	*Sd-Sf*	*Sd-Sf*	*WPH*	*Sd-Sf*	*Sd-Sf*	*Sd-Sf*	*Sd-Sf*	MPH	*Sd-Sf*
Specific Leaf Area	*Sd-Sf*	*Sd-Sf*	MPH	MPH	*Sd-Sf*	*Sd-Sf*	*Sd-Sf*	*Sd-Sf*	*Sd-Sf*	*Sd-Sf*	*Sd-Sf*	MPH
Stomatal conductance	MPH	*Sd-Sf*	*Sd-Sf*	*Sd-Sf*	*Sd-Sf*	*Sd-Sf*	MPH	*Sd-Sf*	MPH	*Sd-Sf*	*Sd-Sf*	MPH
Tiller biomass (% AGB)	MPH	MPH	BPH	MPH	*Sd-Sf*	BPH	*Sd-Sf*	*Sd-Sf*	BPH	*Sd-Sf*	*Sd-Sf*	A
Tiller length	BPH	BPH	BPH	BPH	BPH	BPH	BPH	BPH	BPH	BPH	BPH	BPH
Tillers growth rate (TGR)	BPH	BPH	BPH	BPH	MPH	MPH	MPH	MPH	BPH	*Sd-Sf*	BPH	*Sd-Sf*
Water Use Eficiency	*Sd-Sf*	*Sd-Sf*	*Sd-Sf*	*Sd-Sf*	*Sd-Sf*	*Sd-Sf*	*Sd-Sf*	MPH	*Sd-Sf*	*Sd-Sf*	*Sd-Sf*	*Sd-Sf*
Δ rhizome TNC	BPH	BPH	BPH	BPH	BPH	BPH	BPH	BPH	BPH	BPH	BPH	BPH
