# Supplementary material for: Transgressivity in Key Functional Traits Rather Than Phenotypic Plasticity Promotes Stress Tolerance in A Hybrid Cordgrass
Source: Plants (Basel). 2019 Dec 12;8(12):594. doi: 10.3390/plants8120594 (PMC6963473; doi:10.3390/plants8120594)
Supplement: Supplementary file 1 [file plants-08-00594-s001.zip › Table_S2.pdf]

Table S2. Number of individuals of *Spartina densiflora x foliosa* (n = 4) with worst- or best-parent heterosis for 36 traits measured at each treatment combination of salinities (0.5, 10, 20 and 40 ppt) and inundation depths (shallow inundation (SI), 4.4 cm deep; intermediate inundation (II), 35.5 cm deep; deep inundation (DI), 55.0 cm deep). Percentages of individuals are marked in bold.

	0.5SI	10SI	20SI	40SI	SI	0.5II	10II	20II	40II	II	0.5DI	10DI	20DI	40DI	DI	0.5	10	20	40	Total
AGB : BGB	4	4	4	4	**100**	4	4	4	4	**100**	4	4	4	4	**100**	**100**	**100**	**100**	**100**	**100**
Tiller length	4	4	4	4	**100**	4	4	4	3	**94**	4	4	4	4	**100**	**100**	**100**	**100**	**92**	**98**
Δ rhizome TNC	4	4	4	4	**100**	4	4	4	4	**100**	4	4	4	4	**100**	**100**	**100**	**100**	**100**	**100**
Root Mass Ratio	2	2	4	4	**75**	2	2	4	4	**75**	4	4	4	2	**88**	**67**	**67**	**100**	**83**	**79**
Tillers growth rate (TGR)	3	4	3	4	**88**	4	0	1	4	**56**	3	1	3	2	**56**	**83**	**42**	**58**	**83**	**67**
Rhizome TNC	1	2	1	4	**50**	0	3	3	4	**63**	4	1	3	4	**75**	**42**	**50**	**58**	**100**	**63**
Rhizome Nitrogen content	3	3	4	0	**63**	2	0	3	3	**50**	0	0	1	1	**13**	**42**	**25**	**67**	**33**	**42**
Chl *a* content	2	3	0	4	**56**	1	0	0	0	**6**	3	3	0	1	**44**	**50**	**50**	**0**	**42**	**35**
Rhizome C:N ratio	3	2	4	0	**56**	2	0	3	2	**44**	0	0	1	1	**13**	**42**	**17**	**67**	**25**	**38**
Leaf Na content	1	1	3	2	**44**	0	1	3	2	**38**	0	0	1	3	**25**	**8**	**17**	**58**	**58**	**35**
Na excretion	0	0	0	0	**0**	2	0	1	0	**19**	4	1	0	1	**38**	**50**	**8**	**8**	**8**	**19**
Chl. *a* + *b* content	1	3	0	4	**50**	1	0	0	0	**6**	3	3	0	1	**44**	**42**	**50**	**0**	**42**	**33**
Chl *b* content	2	2	0	1	**31**	1	0	0	0	**6**	2	0	0	1	**19**	**42**	**17**	**0**	**17**	**19**
Net photosynthesis rate	0	1	0	1	**13**	0	2	0	2	**25**	1	0	0	2	**19**	**8**	**25**	**0**	**42**	**19**
Proline content	2	2	2	0	**38**	0	0	1	0	**6**	0	0	0	2	**13**	**17**	**17**	**25**	**17**	**19**
Glycinebetaine content	3	2	0	0	**31**	0	2	0	0	**13**	2	0	0	0	**13**	**42**	**33**	**0**	**0**	**19**
Stomatal conductance	1	1	1	0	**19**	1	1	1	0	**19**	0	0	0	2	**13**	**17**	**17**	**17**	**17**	**17**
Root porosity	0	0	2	1	**19**	2	0	0	1	**19**	0	1	0	1	**13**	**17**	**8**	**17**	**25**	**17**
Carotenoids content	0	2	0	1	**19**	0	0	0	0	**0**	2	1	0	1	**25**	**17**	**25**	**0**	**17**	**15**
Specific Leaf Area	2	1	1	0	**25**	0	0	0	0	**0**	2	1	0	0	**19**	**33**	**17**	**8**	**0**	**15**
Chl *a* : Carotenoids ratio	1	0	1	1	**19**	0	0	0	1	**6**	1	0	1	1	**19**	**17**	**0**	**17**	**25**	**15**
Below-ground biomass (BGB)	2	3	1	0	**38**	0	0	0	0	**0**	0	1	0	0	**6**	**17**	**33**	**8**	**0**	**15**
Leaf Rolling	2	2	0	2	**38**	0	0	0	0	**0**	0	0	0	1	**6**	**17**	**17**	**0**	**25**	**15**
Root biomass (% BGB)	0	0	2	1	**19**	0	1	0	0	**6**	1	1	0	0	**13**	**8**	**17**	**17**	**8**	**13**
Leaf biomass (% AGB)	0	0	0	3	**19**	0	0	1	0	**6**	0	0	2	0	**13**	**0**	**0**	**25**	**25**	**13**
Leaf Nitrogen content	3	0	2	0	**31**	0	0	0	0	**0**	0	0	0	1	**6**	**25**	**0**	**17**	**8**	**13**
Leaf C:N ratio	3	0	2	0	**31**	0	0	0	0	**0**	0	0	0	1	**6**	**25**	**0**	**17**	**8**	**13**
Rhizome biomass (% BGB)	0	0	2	1	**19**	0	2	0	0	**13**	1	0	0	0	**6**	**8**	**17**	**17**	**8**	**13**
Tiller biomass (% AGB)	0	1	1	3	**31**	0	0	0	1	**6**	0	0	0	0	**0**	**0**	**8**	**8**	**33**	**13**
Inflorescences biomass (% AGB)	0	4	1	0	**31**	0	0	0	0	**0**	0	0	0	0	**0**	**0**	**33**	**8**	**0**	**10**
Chl *a* : Chl *b* ratio	2	0	1	0	**19**	0	2	0	0	**13**	0	0	0	0	**0**	**17**	**17**	**8**	**0**	**10**
Water Use Eficiency	0	0	0	0	**0**	0	0	1	0	**6**	0	0	1	1	**13**	**0**	**0**	**17**	**8**	**6**
Rhizome porosity	0	0	0	1	**6**	0	0	0	0	**0**	0	0	0	0	**0**	**0**	**0**	**0**	**8**	**2**
Leaf Water content	0	0	0	0	**0**	0	0	0	0	**0**	0	0	0	0	**0**	**0**	**0**	**0**	**0**	**0**
Leaf Carbon content	0	0	0	0	**0**	0	0	0	0	**0**	0	0	0	0	**0**	**0**	**0**	**0**	**0**	**0**
Rhizome Carbon content	0	0	0	0	**0**	0	0	0	0	**0**	0	0	0	0	**0**	**0**	**0**	**0**	**0**	**0**
**Total**	**34**	**36**	**34**	**34**		**20**	**19**	**23**	**24**		**30**	**20**	**20**	**28**						
